# Clinical characteristics of hypoglossal nerve palsy secondary to internal carotid artery dissection: a systematic review and illustrative case

**DOI:** 10.3389/fneur.2026.1816408

**Published:** 2026-06-02

**Authors:** Jee Ho Kang, Sanghyuk Im, Seung Yoon Song

**Affiliations:** 1 Department of Neurosurgery, Yeouido St. Mary’s Hospital, The Catholic University of Korea, Yeongdeungpo-gu, Seoul, Republic of Korea; 2 Department of Neurosurgery, Eunpyeong St. Mary’s Hospital, The Catholic University of Korea, Eunpyeong-gu, Seoul, Republic of Korea

**Keywords:** cranial nerve palsy, diagnostic delay, hypoglossal nerve palsy (HNP), internal carotid artery dissection (ICAD), pseudoaneurysm, systematic review

## Abstract

**Background:**

Hypoglossal nerve palsy (HNP) secondary to extracranial internal carotid artery dissection (ICAD) is a rare but clinically important condition that may be overlooked, particularly in the absence of ischemic lesions on brain imaging. We aimed to systematically characterize its clinical features, diagnostic patterns, treatment strategies, and outcomes.

**Methods:**

A systematic review was conducted in accordance with PRISMA 2020 guidelines and registered in PROSPERO (CRD420251141162). PubMed, Scopus, Web of Science, and Embase were searched without language or date restrictions. Studies reporting adult patients with HNP attributable to ICAD were included. Clinical data, imaging findings, treatment modalities, and outcomes were extracted and analyzed descriptively and exploratorily.

**Results:**

A total of 73 studies comprising 87 patients were included. The mean age was 48.5 years, and 86.2% were male. Isolated HNP was observed in 64.3% of cases, and diagnostic error occurred in 31.0%, frequently leading to delayed management. Most patients were managed medically (89.7%), and overall favorable outcomes were achieved in 65.6% of patients. Surgical or endovascular treatment was performed in a minority of cases (10.3%). The presence of pseudoaneurysm was significantly associated with increased likelihood of surgical or endovascular treatment (*p* = 0.007) and shorter follow-up duration (*p* = 0.015), although overall outcomes did not differ between groups.

**Conclusion:**

Extracranial ICAD presenting as HNP is an uncommon but clinically important condition with a substantial risk of diagnostic delay. Early vascular imaging should be considered in patients with isolated or atypical hypoglossal nerve palsy, even in the absence of ischemic lesions on brain MRI. Most patients achieve favorable outcomes with medical therapy; however, selected patients—particularly those with pseudoaneurysm or persistent or progressive symptoms—may require surgical or endovascular intervention. Further large-scale studies are needed to refine patient selection and optimize management strategies.

**Systematic review registration:**

https://www.crd.york.ac.uk/PROSPERO/view/CRD420251141162, identifier (CRD420251141162).

## Introduction

Hypoglossal nerve palsy (HNP) is an uncommon neurological manifestation that typically presents with tongue weakness, dysarthria, and dysphagia (1). On neurological examination, tongue deviation or atrophy may be observed. These symptoms often prompt clinicians to suspect acute cerebral infarction or intracranial hemorrhage, making brain imaging the initial diagnostic step.

When neuroimaging does not demonstrate evidence of stroke, a broad range of differential diagnoses should be considered (1). Common etiologies include compression by pathological lesions, such as osteophytes (2), cysts (3, 4), or tumor (5, 6), as well as radiotherapy, intraoperative injury (7), trauma (7), infection (7, 8), and immunological or inflammatory disorders including rheumatoid arthritis (9). Many of these causes can be suspected based on the patient’s history and clinical course. In contrast, vascular causes of HNP may be overlooked unless clinicians maintain a high index of suspicion and perform appropriate vascular imaging.

Internal carotid artery dissection (ICAD) is a recognized cause of ischemic stroke and less commonly, hemorrhagic presentation. Intracranial ICAD may present with vascular occlusion or subarachnoid hemorrhage, whereas extracranial ICAD is relatively rare, accounting for 1–2% of all strokes (10). Nevertheless, extracranial ICAD is an important cause of ischemic stroke in younger patients, contributing to 10–25% of cases under the age of 50 (10). Clinical presentations are diverse and may include head or neck pain, Horner’s syndrome, cranial nerve palsy, and pulsatile tinnitus (1, 10–12).

Extracranial ICAD presenting solely as isolated HNP without ischemic events is exceedingly rare. In particular, when brain magnetic resonance imaging (MRI) shows no ischemic lesions, additional vascular imaging may be delayed or omitted, potentially leading to diagnostic delay or missed diagnosis. Because of this atypical presentation, patients may experience prolonged uncertainty before receiving a definitive diagnosis. Recognizing this entity is therefore critical for timely evaluation and management.

At our institution, we encountered a patient with isolated HNP caused by extracranial ICAD. While reviewing the literature to guide management, we found that most available evidence was limited to individual case reports based on isolated clinical experiences, and that previously published literature reviews had included fewer than 20 cases. Consequently, comprehensive and systematic data regarding diagnostic approaches, treatment strategies, and clinical outcomes remain limited.

To address this gap, we present our institutional case as an illustrative example and conducted a systematic review to synthesize the available evidence. Specifically, we aimed to clarify the clinical features, diagnostic pathways, therapeutic strategies, and outcomes of patients with HNP secondary to extracranial ICAD. By providing a structured synthesis of published cases together with an illustrative case, this study seeks to improve clinical recognition, inform management decisions, and identify areas for future investigation.

## Methods

This systematic review was conducted in accordance with the PRISMA 2020 guidelines, and the protocol was prospectively registered in PROSPERO (registration number: CRD420251141162).

### Search strategy and eligibility criteria

A comprehensive search was performed in PubMed, Scopus, Web of Science, and Embase on September 1, 2025. The detailed search strategies for each database are presented in Supplementary Table 1. No restrictions were applied with regard to language or publication date.

Studies were eligible if they reported adult patients (≥18 years) with hypoglossal nerve palsy attributable to internal carotid artery dissection (ICAD), either isolated or in combination with other cranial nerve palsies. Studies were excluded if hypoglossal nerve palsy was caused by other etiologies such as tumors, major trauma, stroke, or infections; if ICAD was not demonstrated; if vascular abnormalities other than ICAD (e.g., vertebral artery dissection) were described; or if acute cerebral infarction was confirmed on diffusion-weighted MRI.

### Study selection

The database search identified 119 records from PubMed, Scopus, Web of Science, and Embase. In addition, 25 records were identified through manual screening of reference lists from relevant review articles and included studies. After removal of 44 duplicates, 100 records remained for screening. These records were independently screened by two reviewers at the title/abstract and full-text levels, with discrepancies resolved by consensus.

A total of 27 records were excluded for the following reasons: acute cerebral infarction (*n* = 11), vertebral artery involvement (*n* = 4), review articles without extractable individual patient data (*n* = 2), conference abstracts (oral presentations or poster formats) (*n* = 7), and unavailable full texts (*n* = 3).

**Figure 1:**
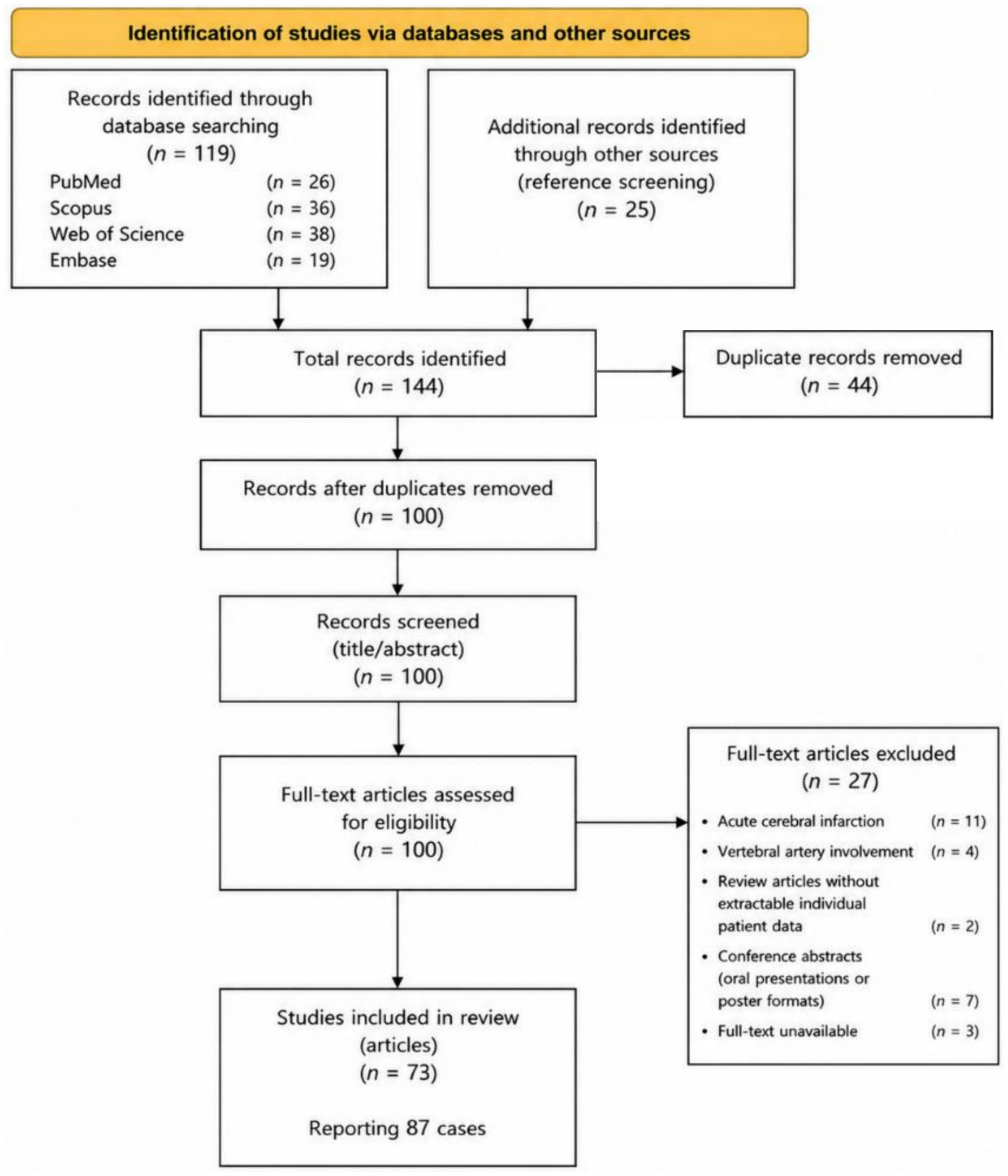
PRISMA flow diagram of study selection process.

Finally, 73 articles reporting 87 cases were included and are listed in the reference section and summarized in Supplementary Table 2. The selection process is summarized in Figure 1.

### Data extraction and collection

Two reviewers independently extracted data using a predefined Excel sheet, and disagreements were resolved by consensus. The following variables were collected: age, sex, trauma history, lesion location, coexistence of pseudoaneurysm with ICAD, anatomical segment of ICAD, and presence of multiple cranial nerve palsies including the hypoglossal nerve. Trauma was defined as a minor physical event preceding symptom onset, excluding major trauma such as fractures (e.g., heavy lifting, sports activity, cervical massage). Both patient-reported symptoms and physician-documented signs were recorded.

Imaging modalities used for diagnosis were noted. Treatments were categorized as medical treatment (medication only) or surgical treatment, the latter including both open surgery and endovascular interventions. Treatment response was classified as complete recovery (full return to baseline), almost complete recovery (nearly full return to baseline), partial recovery (improvement with residual symptoms), or no recovery (no improvement). For analysis, outcomes were dichotomized as good outcome (complete or almost complete recovery) and poor outcome (partial or no recovery).

The follow-up period was defined as the interval from symptom onset to the last recorded clinical visit, and the recovery period was defined as the time from symptom onset to the first documented sign of improvement. For this review, diagnostic error was defined as either an initial misdiagnosis resulting in inappropriate treatment or a delay in diagnosing ICAD, during which patients did not receive appropriate management until later evaluation in another department. Because of heterogeneous reporting across studies, no strict time threshold was applied; diagnostic error was identified qualitatively based on clinical descriptions. When ambiguity existed, cases were conservatively classified as having no diagnostic error.

Most patient-level data were available; however, in some cases, treatment methods, recovery status, or recovery times were incomplete. These were treated as missing values and excluded from statistical analyses.

### Risk of bias assessment

Risk of bias was assessed using the Joanna Briggs Institute (JBI) critical appraisal checklist for case reports. Each included study was evaluated based on predefined criteria, and the results were summarized descriptively. Given the nature of case reports, the assessment focused on overall reporting quality rather than formal quantitative scoring. Detailed results are provided in Supplementary Table 3.

### Statistical analysis

Data were analyzed using SPSS (version 23, IBM Corp., Armonk, NY, United States). Continuous variables were expressed as mean ± standard deviation or median with interquartile range, depending on distribution. Categorical variables were summarized as frequencies and percentages. Comparisons were made using the chi-square or Fisher’s exact test for categorical variables, and the *t*-test or Mann–Whitney *U* test for continuous variables. Logistic regression analysis was performed to explore potential associations between clinical variables and diagnostic error and clinical outcome. Given the heterogeneity and limited sample size, these analyses were considered exploratory. A *p*-value < 0.05 was considered statistically significant.

In addition, an institutional case is presented separately as an illustrative example to provide clinical context and was not included in the systematic review dataset or statistical analyses.

## Results

This systematic review was conducted in accordance with the PRISMA 2020 guidelines. The initial search identified 135 articles; after removing duplicates and applying exclusion criteria, 73 articles comprising 87 cases were included. The baseline characteristics of the patients are summarized in Table 1. The mean age was 48.5 ± 8.3 years, and males were predominant (75 patients, 86.2%). A history of trauma was reported in 22 patients (25.3%). The lesion was more frequently located on the left side (66 patients, 75.8%), and 30 patients (35.5%) had a coexisting pseudoaneurysm. Involvement of the cervical and petrous segments of the internal carotid artery was observed in 14 patients (16.1%). Clinically, isolated hypoglossal nerve palsy was present in 56 patients (64.3%), while multiple cranial nerve palsies were reported in 31 patients (35.7%), most often affecting the glossopharyngeal, vagus, and accessory nerves. A diagnostic error was documented in 27 patients (31.0%).

**Table 1:** Baseline demographic and clinical characteristics.

Category	Value
Age (year)	48.5 ± 8.3
Sex	
Male	75 (86.2%)
Female	12 (13.8%)
History of trauma	
No	65 (74.7%)
Yes	22 (25.3%)
Lesion side	
Left	66 (75.8%)
Right	21 (24.2%)
Pseudoaneurysm (PA)	
Without PA	57 (65.5%)
With PA	30 (35.5%)
Involved ICA segment	
Cervical segment	73 (83.9%)
Cervical, petrous segment	14 (16.1%)
Involved cranial nerve (CN)	
Isolated hypoglossal nerve (HNP)	56 (64.3%)
Multiple CN involvement	31 (35.7%)
Diagnostic error	27 (31.0%)
Follow-up periods (week)	23.4 ± 30.7
Recovery periods (week)	12.0 ± 21.8
Treatment response	
Complete	35 (40.2%)
Almost complete	22 (25.3%)
Partial	20 (23%)
No recovery	2 (2.3%)
Outcome	
Good	57 (65.5%)
Poor	22 (25.3%)

Data are presented as mean ± standard deviation or *n* (%).

ICA, internal carotid artery; CN, cranial nerve; HNP, hypoglossal nerve palsy; PA, pseudoaneurysm.

Clinical symptoms and signs at initial presentation were analyzed (Table 2). Most patients exhibited multiple findings. The most common symptoms were tongue weakness (76.1%), dysarthria (65.6%), and dysphagia (64.8%). Tongue swelling (11.4%), a nonspecific symptom, was also reported in some cases and may have contributed to diagnostic delay or misdiagnosis. The most frequent clinical sign was tongue deviation (84.1%), followed by tongue atrophy (28.4%), soft palate deviation (26.1%), and Horner syndrome (19.3%). Headache (54.5%) and jaw or neck pain (52.2%), which are commonly associated with internal carotid artery dissection, were observed in approximately half of the patients. Overall, tongue weakness and tongue deviation emerged as the most characteristic clinical features of hypoglossal nerve palsy caused by internal carotid artery dissection.

**Table 2:** Clinical symptoms and signs of patients with hypoglossal nerve palsy secondary to internal carotid artery (ICA) dissection.

Symptom or sign	Frequency	Percentage (%)
Symptoms		
Tongue weakness	67	76.1
Dysarthria	58	65.9
Dysphagia	57	64.8
Headache	48	54.5
Jaw or neck pain	36	52.2
Hoarseness	28	31.8
Tongue swelling	10	11.4
Signs		
Tongue deviation	74	84.1
Tongue atrophy	25	28.4
Soft palate deviation	23	26.1
Horner syndrome	17	19.3

Imaging modalities used for the diagnosis of ICA dissection with hypoglossal nerve palsy included MRI, computed tomography (CT), digital subtraction angiography (DSA), and ultrasonography. MRI was the most frequently used technique (72 cases, 81.8%), followed by CT (51 cases, 58.0%) and DSA (42 cases 47.7%). In most patients, more than one modality was performed, and the demonstration of an intramural hematoma on MRI served as the principal evidence for confirmation of dissection. In cases with a coexisting pseudoaneurysm, DSA was essential for precise lesion evaluation, and 7 cases proceeded to neuro-interventional treatment. Ultrasonography was performed in 20.5% (18 cases) of cases but provided limited diagnostic value.

Treatment of ICA dissection was divided into medical and surgical approaches (Table 3). Medical treatment was administered in 78 patients (89.7%), whereas surgical treatment was performed in 9 patients (10.3%). Among medical strategies, anticoagulation was most frequently used (26 patients, 29.9%), followed by antiplatelet therapy (21 patients, 24.1%) and conservative management (21 patients, 24.1%). A combination of anticoagulation and antiplatelet agents, or sequential use as bridge therapy, was reported in 10 patients (11.5%). Surgical treatment consisted of open surgery in 2 patients (2.3%) and neuro-intervention in 7 patients (8.0%). Neuro-interventional procedures included coil embolization (2 patients) and stent insertion (5 patients).

**Table 3:** Treatment modalities of patients.

Treatment modalities	*n*	Percentage (%)
Medical treatment	78	89.7
AC	26	29.9
AP	21	24.1
AC + AP	10	11.5
Conservative treatment	21	24.1
Surgical treatment	9	10.3
Open surgery	2	2.3
Neuro-Intervention	7	8
Total	87	100

AC, Anticoagulant; AP, Antiplatelet.

Treatment response was assessed by recovery status. Complete recovery was observed in 35 patients (40.2%), almost complete recovery in 22 patients (25.3%), partial recovery in 20 patients (23.0%), and no recovery in 2 patients (2.3%). When dichotomized, a favorable outcome (complete or almost complete recovery) was achieved in 57 patients (65.6%). The mean follow-up period was 23.4 ± 30.7 weeks, and the mean recovery period was 12.0 ± 21.8 weeks (Table 4).

**Table 4:** Follow-up and recovery periods according to treatment modalities.

Treatment modalities	Patients	Follow-up period (week)	Recovery period (week)
Medical group	67	23.8 ± 29.3	12.8 ± 23.0
AC	19	23.4 ± 21.3	9.2 ± 8.8
AP	20	26.0 ± 37.7	15.8 ± 34.9
AC with AP	10	18.7 ± 30.4	8.2 ± 5.4
Conservative treatment	18	24.5 ± 26.3	15.9 ± 23.6
Surgical group	9	20.2 ± 41.5	5.3 ± 3.9
Neuro-Intervention	7	7.1 ± 4.7	4.8 ± 4.3
Open surgery	2	66.1 ± 90.7	7.2 ± 2.1
Total	76	23.4 ± 30.7	12.0 ± 21.8
*p*-value*		**0.081**	0.175

Data are presented as mean ± standard deviation.

*p-values were calculated for comparisons between the medical and surgical groups.

AC, anticoagulant; AP, antiplatelet.

Bold value indicates a *p*-value between 0.05 and 0.10, which was interpreted as indicating a statistical trend.

Follow-up period, recovery period, treatment response, and clinical outcome were analyzed according to treatment modality. Cases with missing information were excluded. As shown in Table 4, the neuro-intervention group demonstrated a shorter follow-up period (7.1 ± 4.7 weeks) and recovery period (4.8 ± 4.3 weeks) compared with other groups, but the differences were not statistically significant. When patients were classified into surgical and medical groups, the surgical group showed a shorter follow-up period (20.2 ± 41.5 weeks, *p* = 0.081) and recovery period (5.3 ± 3.9 weeks, *p* = 0.175), with only a trend observed for follow-up duration.

Table 5 summarizes treatment response and outcome. A favorable outcome was achieved in approximately 72.5% of all patients. In most treatment groups, more than 80% achieved complete or almost complete recovery, whereas the anticoagulation group (66.7%) and conservative treatment group (57.9%) showed lower rates. In contrast, favorable outcomes were observed in 100% of surgically treated patients and 85.7% of those undergoing neuro-intervention. When grouped as surgical versus medical treatment, favorable outcomes were more frequent in the surgical group (88.9% vs. 70.4%), although the difference was not statistically significant (*p* = 0.432).

**Table 5:** Treatment response and outcome according to treatment modalities.

Treatment modality	Treatment response				Outcome		
	Complete	Almost complete	Partial	No recovery	Good	Poor	Total
Medical group							
AC	10 (47.6%)	4 (19.0%)	7 (33.3%)	0	14 (66.7%)	7 (33.3%)	21
AP	9 (42.9%)	8 (38.1%)	4 (19.0%)	0	17 (81%)	4 (19%)	21
AC + AP	5 (50.0%)	3 (30.0%)	2 (20.0%)	0	8 (80%)	2 (20%)	10
CT	7 (36.8%)	4 (21.1%)	6 (31.6%)	2 (10.5%)	11 (57.9%)	8 (42.1%)	19
Surgical group							
Neuro-Intervention	3 (42.9%)	3 (42.9%)	1 (14.3%)	0	6 (85.7%)	1 (14.3%)	7
Open surgery	1 (50%)	1 (50%)	0	0	2 (100%)	0	2
Total	35 (43.8%)	23 (28.7%)	20 (25.0%)	2 (2.6%)	58 (72.5%)	22 (27.5%)	80
*P*-value*					0.432		

*P-value represents comparison of outcome distribution (good vs. poor outcome) between the medical and surgical groups using the chi-square or Fisher’s exact test as appropriate. Data are presented as *n* (%).

AC, anticoagulant; AP, antiplatelet; AC+AP, anticoagulant with antiplatelet; CT, conservative treatment.

Patients with ICA dissection were divided into two subgroups: Dissection with pseudoaneurysm (DWPA) group and Dissection without pseudoaneurysm (DWOA) group (Table 6). A total of 31 patients (35.7%) had an associated pseudoaneurysm.

**Table 6:** Comparison between patients with ICA dissection only and those with pseudoaneurysm.

Variable	DWOA (*n* = 57)	DWPA (*n* = 30)	*p*-value
Age (years)	49.5 ± 8.5	46.9 ± 7.8	0.17
Sex (male)	51 (89.5%)	24 (80.0%)	0.223
History of trauma	13 (22.8%)	9 (30.0%)	0.463
Left-sided lesion	44 (77.2%)	22 (73.3%)	0.669
Cervical ICA involvement	45 (78.9%)	28 (93.3%)	0.083
Multiple cranial nerve involvement	19 (33.3%)	12 (40.0%)	0.639
Follow up period (week)	27.9 ± 35.0	14.2 ± 16.0	**0.015**
Recovery period (week)	14.5 ± 25.8	6.7 ± 7.3	0.141
Surgical treatment	2 (3.5%)	8 (26.7%)	**0.003**
Good outcome	39 (68.4%)	18 (60.0%)	0.248

Data are presented as mean ± standard deviation or *n* (%). *P*-values were calculated using the Student’s *t*-test or chi-square/Fisher’s exact test as appropriate.

DWOA, dissection without aneurysm; DWPA, dissection with pseudoaneurysm; ICA, internal carotid artery.

Bold values indicate *P*-values < 0.05, which were considered statistically significant.

Multiple cranial nerve involvement was observed in 12 patients (38.7%) in the DWPA group and 19 patients (61.3%) in the DWOA group, with no significant difference between groups (*p* = 0.646). Surgical treatment was performed significantly more often in the DWPA group (*p* = 0.007), suggesting that the presence of pseudoaneurysm influenced therapeutic decision-making.

The mean follow-up period was shorter in the DWPA group (14.2 ± 16.0 weeks) than in the DWOA group (27.9 ± 35.0 weeks, *p* = 0.015). The recovery period was also shorter in the DWPA group (6.7 ± 7.3 weeks vs. 14.5 ± 25.8 weeks), although this difference was not statistically significant. No significant differences in overall outcomes were identified between the two groups.

We examined factors that might influence recovery from hypoglossal nerve palsy, with a focus on diagnostic error. Diagnostic error was defined as an initial misdiagnosis leading to inappropriate management or a delayed diagnosis without appropriate treatment until subsequent evaluation in another department. It was identified in 31% (27 patients). Patients were divided into a diagnostic error group and a no error group for comparison (Table 7).

**Table 7:** Comparison between patients with and without diagnostic error.

Variable	Diagnostic error group (*n* = 27)	No error group (*n* = 57)	*p*-value
Age (years)	48.9 ± 9.6	48.3 ± 7.7	0.796
Sex (male)	21 (77.8%)	54 (94.7%)	0.126
History of trauma	7 (25.9%)	15 (26.3%)	0.927
Left-sided lesion	19 (70.4%)	48 (84.2%)	0.422
ICA dissection with pseudoaneurysm	10 (37.0%)	20 (35.1%)	0.737
Cervical ICA involvement	23 (85.2%)	50 (87.7%)	0.828
Multiple CN involvement	17 (63.0%)	39 (68.4%)	0.854
Follow up period (week)	26.1 ± 26.4	22.2 ± 32.7	**0.058**
Recovery period (week)	14.1 ± 22.8	11.3 ± 21.7	0.214
Surgical treatment	1 (3.7%)	8 (14.0%)	0.116
Good outcome	17 (63.0%)	40 (70.2%)	0.575

Data are presented as mean ± standard deviation or *n* (%). *P*-values were calculated using the Student’s *t*-test or chi-square/Fisher’s exact test as appropriate.

ICA, internal carotid artery; CN, cranial nerve.

Bold value indicates a *P*-value between 0.05 and 0.10, which was interpreted as indicating a statistical trend.

There were no significant differences in baseline demographics, treatment modality (*p* = 0.263), or final outcome (*p* = 0.594) between groups. Follow-up duration and recovery period tended to be longer in the error group but were not statistically significant. Notably, the follow-up duration was 26.1 ± 26.4 weeks in the error group versus 22.2 ± 32.7 weeks in the no error group, showing a near-significant trend (*p* = 0.058). Although statistical significance was not reached, the 31% error rate indicates potential clinical relevance that warrants attention.

### Illustrative case

**Figure 2:**
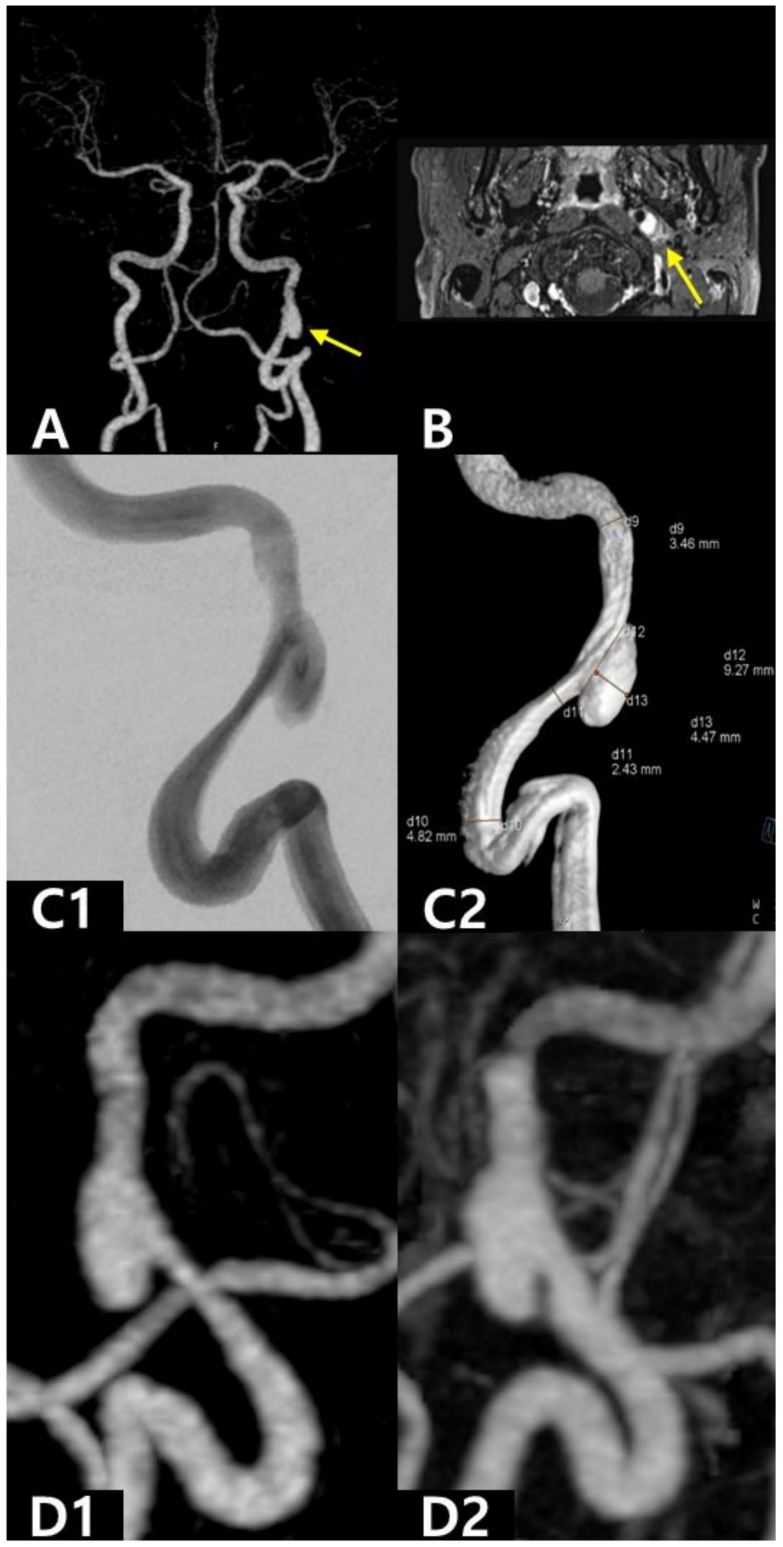
Multimodal imaging findings of the illustrative case. **(A)** Initial CTA showing focal dilatation in the cervical segment of the left ICA (arrow). **(B)** Vessel wall MRI demonstrating intramural hematoma (arrow), consistent with arterial dissection. **(C1)** DSA showing luminal irregularity with focal dilatation, consistent with a dissecting aneurysm. **(C2)** Three-dimensional reconstruction illustrating the morphology of the lesion. **(D1)** Initial CTA. **(D2)** Follow-up CTA at 6 months demonstrating marked improvement of the arterial stenosis compared with the initial study.

A 42-year-old man presented with dysarthria of 5 days’ duration and left posterior neck pain that began 7 days prior. Neurological examination revealed leftward tongue deviation, consistent with hypoglossal nerve palsy, without other deficits. Brain MRI showed no acute infarction or hemorrhage. CTA demonstrated a focal dilatation in the cervical segment of the left ICA. Vessel wall MRI revealed intramural hematoma, consistent with arterial dissection. DSA demonstrated luminal irregularity with focal dilatation, consistent with a dissecting aneurysm (Figure 2).

The patient was managed conservatively with dual antiplatelet therapy, antihypertensive medication, and statin therapy. At 2 months, his symptoms had markedly improved, and at 6 months, they had completely resolved. Follow-up CTA showed significant improvement of the arterial stenosis. This case highlights that extracranial ICAD may present as isolated HNP without radiographic evidence of cerebral infarction.

## Discussion

Cervical internal carotid artery (ICA) dissection accounts for 5–25% of ischemic strokes in patients younger than 45 years, with the peak incidence occurring in the fifth decade of life (1, 13–16). The annual incidence in the general population is estimated at 2.5–3 per 100,000 persons (17). Clinical manifestations vary considerably and often include local signs such as head or neck pain, Horner’s syndrome, cranial nerve palsies, and pulsatile tinnitus, which typically precede ischemic stroke (10).

Patients with isolated hypoglossal nerve palsy may present with dysphagia, dysarthria, tongue atrophy, weakness, or deviation (1). Approximately half of all hypoglossal nerve impairments are attributed to tumors; however, 3–15% are idiopathic, with no identifiable lesion even after advanced diagnostic evaluation (1, 7, 8).

### Pathophysiology of hypoglossal nerve palsy caused by extracranial ICA dissection

The hypoglossal nerve exits the skull through the hypoglossal canal and enters the carotid sheath. It courses anteromedial to the ICA down to the level of C2, where it turns posterolateral, crossing the ICA before joining the lingual artery toward the tongue. Because of this close anatomical relationship, abnormalities of the ICA within the carotid sheath can lead to hypoglossal nerve injury.

The manifestations of ICA dissection depend on the arterial wall layer involved (18). Two main patterns are described: subintimal dissection, usually the most common, caused by an intimal tear and false lumen formation with anterograde blood flow, leading to stenosis or occlusion without external expansion; and subadventitial dissection, which originates from rupture of the vasa vasorum and produces an outward hematoma, compressing adjacent structures (16, 19–24). Hypoglossal nerve palsy due to ICA dissection is most often attributed to subadventitial hematoma. In our review, however, many cases showed overlapping features of luminal narrowing and external expansion. Progressive subadventitial hematoma may evolve into a pseudoaneurysm, and in some cases ICA luminal narrowing was also present. Depending on hematoma size and location, additional involvement of other lower cranial nerves or sympathetic fibers was observed.

Nerve damage develops through two principal mechanisms: direct compression or ischemia secondary to disruption of the vasa nervorum (1, 25). Occlusive ICA disease may result from vasospasm, thrombosis, aneurysm, mural fibrosis, contusion, or most commonly an intimal tear (26, 27).

### Clinical characteristics of ICA dissection presenting with hypoglossal nerve palsy

Our systematic review identified several consistent clinical features of ICA dissection presenting with hypoglossal nerve palsy. Isolated hypoglossal nerve involvement was the most common presentation, although approximately one-third of patients exhibited additional cranial nerve deficits. The condition was strongly male-predominant (86.4%) and more frequently affected the left side (76.1%). Most dissections were confined to the cervical segment, but some extended into the petrous ICA. Pseudoaneurysm formation was present in about one-third of cases.

Clinical symptoms were variable, but tongue weakness, dysarthria, and dysphagia were the predominant manifestations. In contrast, symptoms typically associated with ICA dissection—such as headache and jaw or neck pain—were reported in only about half of patients. On physical examination, tongue deviation was the most reliable clinical sign, whereas tongue atrophy was uncommon in the early stage. Finally, diagnostic error or delayed diagnosis was observed in nearly 30% of patients, underscoring the diagnostic challenges associated with this rare presentation.

### Imaging modalities

The MRI is the most useful modality for detecting intramural hematoma and assessing ICA wall diameter to evaluate stenosis (1, 28). In cases with pseudoaneurysm, MRI can also demonstrate intimal flaps (28). High-resolution MRI provides additional value by visualizing not only the vessel but also adjacent cranial nerves, thereby clarifying the cause of associated findings such as Horner’s syndrome or soft palate deviation that cannot be explained by hypoglossal nerve palsy alone. Reported diagnostic performance is high, with MRI showing 84% sensitivity and 99% specificity, and MR angiography (MRA) reaching 95% sensitivity and 99% specificity (29).

Computed tomography angiography (CTA) is another primary diagnostic tool for ICA dissection and intramural hematoma (13, 30, 31). It is particularly useful in emergency settings or trauma-related cases, where MRI may be less feasible (32). CTA also demonstrates strong diagnostic accuracy, with reported sensitivity ranging from 74 to 98% and specificity from 84 to 100% (29). In addition, CT and MRI contribute to the evaluation of sequelae such as cerebral ischemia, muscular edema, and atrophy.

The DSA is not routinely required for diagnosing ICA dissection, but it remains the gold standard for evaluating pseudoaneurysms (33). DSA is more invasive than other imaging modalities, yet it is indispensable when severe stenosis or pseudoaneurysm progression is present, as it guides decisions regarding neuro-intervention.

Ultrasonography is noninvasive and easily performed (1), but its diagnostic accuracy is limited; thus, it plays only a supportive role in the diagnostic work-up.

### Characteristics of ICA dissection patients with pseudoaneurysm

Extracranial internal carotid artery (ICA) aneurysms account for only about 1% of all aneurysmal disease, and within this group, 6–12% are bilateral variants (29, 33). Among patients with ICA dissection, pseudoaneurysms have been reported in approximately 12–17% of cases, representing a serious complication of arterial wall injury. These pseudoaneurysms typically develop within 5 years of the initial dissection (12, 34). In general, the presence of a pseudoaneurysm indicates more advanced or severe disease compared with dissection alone. Theoretically, because of the restricted space within the carotid sheath, pseudoaneurysms may exacerbate symptoms and hinder recovery. However, in the present study, the presence of a pseudoaneurysm did not show a statistically significant association with outcomes (*p* = 0.31) Due to the high risk of embolism and secondary cerebral ischemia, surgical treatment is often recommended, either through endovascular techniques or open resection with grafting (33). In our cohort, one patient underwent open surgery, six received neuro-interventional treatment, and the remaining 24 were managed medically. While acute infarction necessitates consideration of interventional treatment, in cases presenting only with lower cranial nerve palsy without cerebral ischemia, therapeutic decisions should be made more cautiously. Interestingly, patients with pseudoaneurysm had a shorter follow-up period compared with those with dissection alone, which may reflect earlier surgical intervention in this subgroup but does not necessarily translate into better long-term outcomes.

### Diagnostic errors and their clinical implications

In this study, diagnostic errors were identified in 27 patients (30.7%), representing a substantial proportion of cases. Such errors frequently led to delayed treatment and prolonged follow-up periods. Several patients were initially evaluated in the otolaryngology department, where neither physical nor imaging examinations revealed an obvious cause, resulting in delayed management.

Most patients presented with tongue weakness, dysarthria, and dysphagia—symptoms that should raise suspicion for a possible cerebrovascular event. However, in many cases, the absence of acute ischemic lesions on intracranial MRI diffusion sequences led clinicians to exclude vascular pathology. Furthermore, some patients had non-specific symptoms such as tongue swelling or hoarseness, which contributed to diagnostic uncertainty. Tongue swelling, in particular, was observed in 10 patients, among whom the diagnostic error rate was notably high (70%, 7/10). Although the number of cases was small, this finding suggests that non-specific symptoms may substantially contribute to diagnostic delay. Additionally, fewer than 50% of patients reported headache or jaw/neck pain—symptoms commonly associated with ICA dissection—likely reducing clinical suspicion of a vascular etiology.

**Figure 3:**
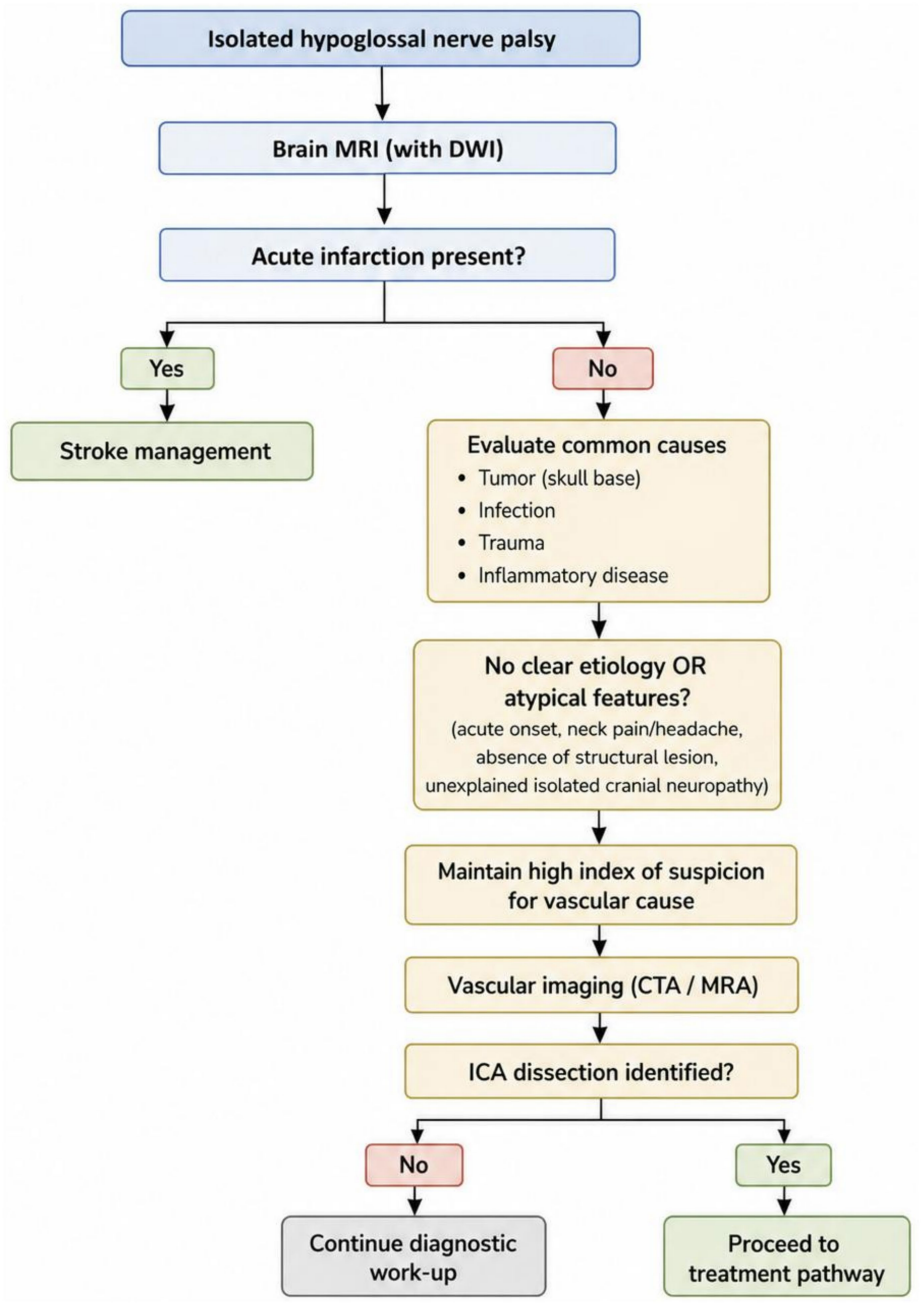
Proposed diagnostic pathway for isolated hypoglossal nerve palsy with consideration of vascular etiology. This algorithm emphasizes the need to consider vascular etiologies, particularly internal carotid artery dissection, when no clear alternative cause is identified.

Therefore, clinicians should maintain a high level of suspicion for cervical ICA dissection when encountering isolated or atypical hypoglossal nerve palsy, even in the absence of acute ischemic findings on MRI. Recognizing these atypical features and pursuing timely vascular imaging may help reduce missed or delayed diagnoses in clinical practice (Figure 3).

### Treatment approaches

The management of ICA dissection can be broadly divided into medical and surgical strategies. In general, the treatment of craniocervical artery dissection begins with antiplatelet or anticoagulation therapy to reduce the risks of thrombosis, embolization, and ischemia (35). When extracranial dissection is accompanied by a pseudoaneurysm, endovascular or surgical intervention may be indicated if neurological symptoms remain refractory to medical therapy and/or if the dissection or pseudoaneurysm continues to expand (34). However, because none of the patients in our study presented with acute cerebral infarction, there are few established treatment guidelines specifically applicable to this cohort.

In our review, treatment modalities included anticoagulant therapy, antiplatelet therapy, combination therapy, surgical procedures, and neuro-intervention. The number of patients within each treatment group was small, which limited the ability to determine superiority of one strategy over another. Nonetheless, the publication year of the included reports revealed temporal changes in treatment patterns. Anticoagulation was the earliest and most widely applied approach, primarily using heparin followed by warfarin at discharge, but reports of anticoagulant monotherapy disappeared after 2017. Antiplatelet monotherapy has been described since 2011 and continues to be a common strategy, most often with dual therapy (aspirin and clopidogrel) during hospitalization, followed by monotherapy at discharge. Combination therapy with both anticoagulants and antiplatelets has also been reported, typically involving inpatient anticoagulation with a switch to antiplatelet therapy before or after discharge. This approach has been in use since 1993 and continues to be described in recent literature.

Neuro-intervention has become increasingly prominent in recent years. Because the dissected ICA wall is highly vulnerable to stenosis or thrombus formation, stent placement may be performed to stabilize the vessel wall (33). For pseudoaneurysms that fail to regress or continue to cause symptoms, coil embolization can be considered (34). More recently, flow-diverter stents have been applied in the extracranial ICA to obliterate pseudoaneurysms (36). These endovascular approaches have generally yielded favorable outcomes, with marked improvement in both recovery period and overall clinical results.

In our cohort, patients who ultimately underwent intervention had initially been managed with antiplatelet or anticoagulant therapy. Because their symptoms failed to improve, treatment was escalated to intervention, typically around 4 weeks after symptom onset. Remarkably, all patients demonstrated rapid clinical improvement within 3 days of the procedure, achieving almost complete or complete recovery. This does not imply that intervention should be considered the first-line therapy, as many patients managed medically also achieved favorable outcomes, and no statistically significant difference in outcomes was found between medical and interventional groups (*p* = 0.432). Nevertheless, neuro-intervention should be considered in the following scenarios: (1) lack of improvement despite adequate medical therapy, (2) persistence of symptoms beyond 4 weeks, (3) disabling lower cranial nerve palsy symptoms such as dysarthria or dysphagia with risk of aspiration pneumonia, and (4) pseudoaneurysm ≥10 mm in size or rapid enlargement within 1–2 weeks.

**Figure 4:**
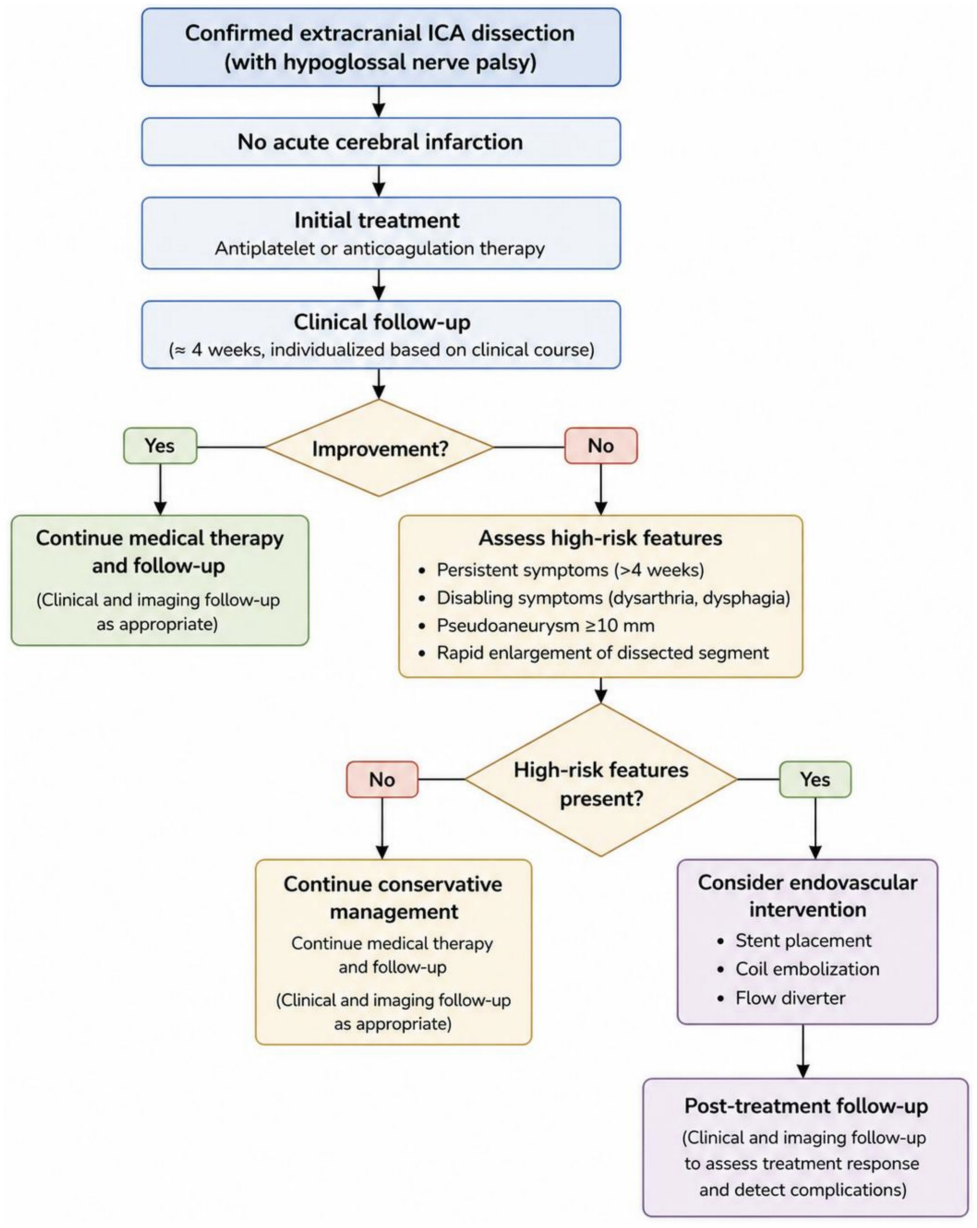
Proposed treatment algorithm for extracranial internal carotid artery dissection presenting with hypoglossal nerve palsy. The algorithm recommends initial conservative management, with consideration of endovascular intervention in patients with persistent symptoms or high-risk features.

These findings highlight possible indications for neuro-intervention in ICA dissection with pseudoaneurysm, but their clinical utility should be confirmed by prospective studies before they can be incorporated into formal guidelines (Figure 4).

### Prognosis

According to previously published small-scale studies, approximately 72% of patients eventually achieve complete resolution of symptoms (10). Hypoglossal nerve palsy related to spontaneous ICA pseudoaneurysm may persist for up to 3 months after onset; however, most cases resolve completely within several months (33).

In our review, favorable outcomes were observed in more than 80% of patients who underwent treatments other than anticoagulation or conservative management. Among those treated with anticoagulants or antiplatelets, 75% achieved favorable outcomes. These findings suggest that with appropriate treatment, the majority of patients experience good recovery. The mean follow-up period across cases was 23.4 weeks, while the average recovery period was 12 weeks. Patients undergoing surgical treatment demonstrated shorter follow-up and recovery periods compared with those treated medically; notably, the mean recovery period was 5.3 ± 3.9 weeks, although this difference did not reach statistical significance (*p* = 0.175). Because most surgically treated patients first received 3–4 weeks of medical therapy, the clinical benefit of surgery itself appeared within approximately 1 week.

No recurrences were identified in our cohort. Previous reports indicate that most recurrences occur within the first 4–6 weeks after symptom onset, typically involving serial arterial dissections (37). After 1 month, the recurrence risk is reported to decline to approximately 1% per year (38).

A total of 22 patients (27.5%) experienced poor outcomes, most of whom were classified as partial recovery with persistent tongue weakness, dysarthria, or dysphagia causing functional discomfort. Of these, 21 had received medical treatment. This finding suggests that patients showing delayed or minimal improvement should be carefully monitored, and surgical treatment should be considered when appropriate to reduce the risk of poor outcomes.

To explore potential predictors of favorable recovery, we performed multivariate analysis and assessed the area under the curve (AUC) for possible cut-off values. However, no model demonstrated strong predictive ability. Using 4 weeks as a threshold to predict favorable outcomes also failed to yield statistical significance (*p* = 0.240). These limitations are likely attributable to the small sample size, heterogeneity in clinical presentations, and variability in management strategies across reported cases. Larger, standardized datasets with consistent documentation of treatment protocols and follow-up are needed to clarify the optimal timing for surgical intervention. These findings indicate that most patients achieve favorable recovery with appropriate management, but larger studies are required to clarify prognostic factors and the optimal timing of surgical intervention.

### Limitations

This study is a systematic review based on previously published case reports. Cases were included without restriction on publication year, introducing heterogeneity in diagnostic methods and treatment strategies over time. Imaging modalities such as MRI, CT, and DSA vary in resolution and equipment performance, making it difficult to apply diagnostic error rates consistently. The definition of diagnostic error was based on qualitative assessment of reported clinical information, which may introduce subjectivity and potential misclassification bias.

Furthermore, substantial heterogeneity was observed across included cases in terms of diagnostic approaches, treatment strategies, and follow-up duration. Diagnostic evaluation varied depending on the imaging modality and timing, while treatment decisions ranged from conservative medical therapy to endovascular intervention without standardized criteria. In addition, follow-up periods and outcome assessments were inconsistent among reports, limiting direct comparison between cases. In addition, follow-up periods and outcome assessments were inconsistent among reports, limiting direct comparison between cases. Therefore, the results of the regression analysis should be interpreted with caution.

Because all information was derived from published articles, missing data and inaccurate reporting are possible. Although we searched three databases and used independent review by two authors, some cases may still have been overlooked. Furthermore, temporal changes in treatment strategies limit the ability to interpret favorable outcomes or recovery periods under a uniform standard. While surgical treatment appeared to yield favorable results, the small number of cases and the fact that such interventions are not yet widely standardized necessitate cautious interpretation.

In addition, case-report–based studies are inherently vulnerable to selection bias, as unusual or severe cases are more likely to be published. Consequently, the generalizability of our findings to the broader population with ICA dissection is limited.

### Future directions

Extracranial ICA dissection is rare, and the presentation with isolated lower cranial nerve palsy is even more uncommon. Consequently, no standardized treatment strategy has been established. Although most patients achieve favorable outcomes with anticoagulation or antiplatelet therapy, the optimal regimen and duration remain uncertain and largely depend on individual physician experience. Large-scale, multicenter studies are required to clarify the efficacy of antithrombotic therapy and to establish evidence-based protocols.

Currently, surgical treatment is rarely considered as a first-line option due to limited evidence and the technical challenges posed by the carotid sheath near the skull base. With the recent advancement of endovascular devices, neuro-intervention is increasingly replacing open surgery. Future studies should systematically evaluate the safety and efficacy of these endovascular techniques—including stent placement and flow diverters—through prospective registries and collaborative clinical research.

## Conclusion

This systematic review demonstrates that extracranial ICAD presenting with HNP is a rare but clinically important condition, frequently associated with diagnostic delay. Given that nearly one-third of patients experienced diagnostic error, early vascular imaging should be strongly considered in patients with isolated or atypical HNP, even in the absence of ischemic lesions on brain MRI.

Most patients achieved favorable outcomes with medical therapy, supporting an initial conservative approach. However, the presence of pseudoaneurysm was associated with a higher likelihood of surgical or endovascular treatment, suggesting that selected patients—particularly those with pseudoaneurysm or persistent or progressive symptoms—may require more active consideration of intervention.

Taken together, these findings highlight that the primary clinical challenge lies in timely diagnosis, while optimal management depends on appropriate patient selection. Further large-scale collaborative studies are warranted to refine treatment strategies and better define indications for intervention in this uncommon condition.

## Data Availability

The original contributions presented in the study are included in the article/Supplementary material, further inquiries can be directed to the corresponding author.
