# Supplementary material for: Clinical characteristics of hypoglossal nerve palsy secondary to internal carotid artery dissection: a systematic review and illustrative case
Source: Front Neurol. 2026 Jun 2;17:1816408. doi: 10.3389/fneur.2026.1816408 (PMC13268898; doi:10.3389/fneur.2026.1816408)
Supplement: Supplementary file 1 [file Supplementary_file_1.docx]

Supplementary Table 1. Full search strategies for each database

Search data: September 1, 2025

| Database | Search strategy |
| --- | --- |
| PubMed | "hypoglossal nerve palsy"[Title/Abstract] AND "internal carotid artery dissection"[Title/Abstract] AND ("case reports"[Publication Type] OR "case series"[Title/Abstract] OR "observational study"[Title/Abstract]) |
| Scopus | TITLE-ABS("hypoglossal nerve palsy") AND TITLE-ABS("internal carotid artery dissection") AND TITLE-ABS("case report" OR "case series" OR "observational study") |
| Web of Science | TS=("hypoglossal nerve palsy") AND TS=("internal carotid artery dissection") AND TS=("case report" OR "case series" OR "observational study") |
| Embase | ('hypoglossal nerve palsy':ti,ab OR 'hypoglossal nerve paralysis':ti,ab OR 'twelfth nerve palsy':ti,ab OR 'cranial nerve xii palsy':ti,ab)  AND  ('internal carotid artery dissection':ti,ab OR 'carotid artery dissection':ti,ab OR 'internal carotid dissection':ti,ab OR 'carotid dissection':ti,ab)  AND  ('case report':ti,ab OR 'case reports':ti,ab OR 'case series':ti,ab OR 'observational study':ti,ab) |

Supplementary Table 2. List of included case reports and case series with author names and number of cases

| Number | Year | Author | Journal | Title | Cases |
| --- | --- | --- | --- | --- | --- |
| 1 | 1981 | Bradac G | Neuroradiology | Spontaneous dissecting aneurysm of cervical cerebral arteries. Report of six cases and review of the literature. | 2 |
| 2 | 1982 | Havelius U | Journal of neurosurgery | Carotid fibromuscular dysplasia and paresis of lower cranial nerves (Collect-Sicard syndrome). Case report. | 1 |
| 3 | 1983 | Goodman J.M | Archives of Neurology | Hemilingual paraly sis caused by spontaneous carotid artery dissection. | 1 |
| 4 | 1984 | Hommel M | Revue neurolgique | Paralysis of the hypoglossal nerve caused by 2 aneurysms and a dissecting aneurysm of the internal carotid artery | 2 |
| 5 | 1986 | Goldberg H | Radiology | Cervical internal carotid artery dissecting hemorrhage: Diagnosis using MR. | 1 |
| 6 | 1987 | Mokri B | Stroke | Familial occurrence of spontaneous dissection of the internal carotid artery | 1 |
| 7 | 1987 | Anzola G | The Italian Journal of Neurological Sciences | Lower cranial nerve palsy produced by internal carotid artery dilatation. Report of two cases | 1 |
| 8 | 1988 | Waespe W | Stroke | Lower cranial nerve palsies due to internal carotid dissection. | 1 |
| 9 | 1988 | Lieschke G | Stroke | Spontaneous internal carotid artery dissection presenting as hypoglossal nerve palsy. | 1 |
| 10 | 1989 | Dal Pozzo G | Journal of computuer assisted tomography | Lower cranial nerve palsy due to dissection of the internal carotid artery: CT and MR imaging | 3 |
| 11 | 1990 | Panisset M | Stroke | Multiple cranial neuropathy as a feature of internal carotid artery dissection | 2 |
| 12 | 1990 | Nusynowitz R | Neuroradiology | Pseudoaneurysm of the cervical internal carotid artery with associated hypoglossal nerve paralysis | 1 |
| 13 | 1992 | Pumar J | The European Journal of Medicine | Pseudoaneurysm of the Cervical Carotid Artery with Hypoglossal and Glossopharyngeal Nerve Paralysis. | 1 |
| 14 | 1993 | Sturzenegger M | Journal of Neurology, Neurosurgery & Psychiatry | Cranial nerve palsies in spontaneous carotid artery dissection. | 1 |
| 15 | 1994 | Klossek J | Annals of Otology, Rhinology & Laryngology | Unilateral lower cranial nerve palsies due to spontaneous internal carotid artery dissection. | 1 |
| 16 | 1998 | Boukobza M | Journal of neuroimaging | Internal carotid artery dissection causes hypoglossal nerve palsy: CT, MRI, and angiographic findings | 1 |
| 17 | 1999 | Lecoules S | La Revue de Medecine Interne | Spontaneous dissecting aneurysm of the internal carotid artery. | 1 |
| 18 | 2000 | Ursekar M | Clinical radiology | Hypoglossal Nerve Palsy Due to Spontaneous Dissection of the Internal Carotid Artery | 1 |
| 19 | 2000 | Heckmann J | Cerebrovascular Diseases | Collet-Sicard syndrome due to coiling and dissection of the internal carotid artery. | 1 |
| 20 | 2001 | Guy N | Canadian journal of neurological sciences | Spontaneous internal carotid artery dissection with lower cranial nerve palsy. | 3 |
| 21 | 2001 | Pakdemirli E | Computerized medical imaging and graphics | Spontaneous bilateral internal carotid artery dissection with hypoglossal nerve palsy | 1 |
| 22 | 2001 | Shahab R | Journal of laringology and otology | Isolated hypoglossal nerve palsy due to internal carotid artery dissection | 1 |
| 23 | 2002 | Bravo-Rodríguez F | Revista de Neurologia | Paralysis of cranial nerves as a form of presentation of dissection of the internal carotid artery | 2 |
| 24 | 2002 | Bonkowsky V | European archives of oto-rhino-laryngology | Vertigo and cranial nerve palsy caused by different forms of spontaneous dissections of internal and vertebral arteries | 1 |
| 25 | 2003 | Lindsay F | The Laryngoscope | Subacute Hypoglossal Nerve Paresis with Internal Carotid Artery Dissection | 1 |
| 26 | 2003 | Wessels T | Der Nervenarzt | Isolated cranial nerve palsy secondary to carotid dissection; Isolierte hirnnervenausfälle bei karotisdissektionen | 1 |
| 27 | 2004 | Hafkamp H | European archives of Oto-Rhino-Laryngology and Head & Neck | Unilateral spontaneous dissection of the internal carotid artery presenting as hypoglossal nerve palsy | 1 |
| 28 | 2004 | Dorsey S | Academic emergency medicine | Clinical pearls: Headache and hypoglossal nerve palsy | 1 |
| 29 | 2005 | Knibb J | The British Journal of Radiology | Internal carotid artery dissection presenting with ipsilateral tenth and twelfth nerve palsies and apparent mass lesion on MRI | 1 |
| 30 | 2006 | Olzowy B | European archives of Oto-Rhino-Laryngology and Head & Neck | Bilateral and unilateral internal carotid artery dissection causing isolated hypoglossal nerve palsy: a case report and review of the literature | 2 |
| 31 | 2009 | Kaushik S | Journal of general interal medicine | Spontaneous Dissection of Internal Carotid Artery Masquerading as Angioedema. | 1 |
| 32 | 2009 | Marin L | Arquivos de neuro-psiquiatria | Hypoglossal nerve palsy as the sole manifestation of spontaneous internal carotid artery dissection. | 1 |
| 33 | 2009 | Bezerra M | Arquivos de neuro-psiquiatria | Spontaneous carotid dissection with hypoglossal nerve palsy as residual deficit: the importance of magnetic resonance evaluation. | 1 |
| 34 | 2009 | Battaglia F | Revue neurolgique | Collet-Sicard syndrome after carotid artery dissection | 1 |
| 35 | 2010 | Arnoldner C | Skull Base | Tenth and twelfth nerve palsies in a patient with internal carotid artery dissection mistaken for cervical mass lesion. | 1 |
| 36 | 2010 | Isildak H | Dysphagia | Unusual manifestations of bilateral carotid artery dissection: Dysphagia and hoarseness. | 1 |
| 37 | 2010 | Mathey D | Circulation | Images in cardiovascular medicine. Hypoglossal-nerve palsy caused by carotid dissection. | 1 |
| 38 | 2010 | Freilinger T | Vascular Medicine | Hypoglossal nerve palsy due to internal carotid artery dissection. | 1 |
| 39 | 2010 | Kasravi N | CMAJ | Dissection of the internal carotid artery causing Horner syndrome and palsy of cranial nerve XII | 1 |
| 40 | 2011 | Stübgen J | Ear Nose Throat Journal | Unilateral Macroglossia as Sole Presenting Manifestation of Internal Carotid Artery Dissection. | 1 |
| 41 | 2011 | Mizutani S | Rinsho Shinkeigaku Clinical Neurology | Villaret’s syndrome caused by internal carotid artery dissection. | 1 |
| 42 | 2011 | Peltz E | New England Journal of Medicine | Internal-carotid-artery dissection and cranial-nerve palsies | 1 |
| 43 | 2012 | Okunomiya T | Neurology | Teaching NeuroImages: isolated hypoglossal nerve palsy due to internal carotid artery dissection | 1 |
| 44 | 2012 | De Santis F | Vascular and endovascular surgery | Internal carotid artery dissection after inferior alveolar nerve block for third molar dental care presented as hypoglossal nerve palsy. | 1 |
| 45 | 2013 | Zeleňák K | Cardiovascular and interventional radiology | Treatment of cervical internal carotid artery spontaneous dissection with pseudoaneurysm and unilateral lower cranial nerves palsy by two silk flow diverters. | 1 |
| 46 | 2013 | Riancho J | Journal of Neurology, Neurosurgery & Psychiatry, | Unilateral isolated hypoglossal nerve palsy associated with internal carotid artery dissection. | 1 |
| 47 | 2013 | Pongmoragot J | Neurology | Pearls and oy  sters: carotid dissection with normal arterial lumen | 1 |
| 48 | 2013 | Ferlazzo E | Neuological Sciences | Isolated hypoglossal nerve palsy due to spontaneous carotid artery dissection: a neuroimaging study. | 1 |
| 49 | 2013 | Smith R | BMJ Case Reports | Collet-Sicard syndrome as a result of unilateral carotid artery dissection | 1 |
| 50 | 2014 | Hennings J | Journal of Stroke and Cerebrovascular Diseases | Painless hypoglossal palsy as an isolated symptom of spontaneous carotid dissection. | 1 |
| 51 | 2014 | Fujii H | Journal of Stroke and Cerebrovascular Diseases | Isolated unilateral hypoglossal nerve paralysis caused by internal carotid artery dissection. | 1 |
| 52 | 2015 | Qi B | Oncology Letters | Bilateral dissecting aneurysms of the internal carotid arteries misdiagnosed as skull base tumors: a case report. | 1 |
| 53 | 2016 | Murakami Y | Journal of Neuroendovascular Therapy | Successfully treated with endovascular therapy against lower cranial nerve paresis caused by spontaneous dissection of the cervical internal carotid artery: A case report | 1 |
| 54 | 2017 | Torbus-Paluszczak M | Neurologia I Neurochirugia Polska | Hypoglossal nerve palsy in the course of dissection of the internal carotid arteries - Case reports. | 2 |
| 55 | 2017 | Cruciata G | Radiology case reports | Internal Carotid Artery Dissection and Pseudoaneurysm Formation with Resultant Ipsilateral Hypoglossal Nerve Palsy | 1 |
| 56 | 2017 | Joshi P | Practical Neurology | Isolated hypoglossal nerve palsy due to internal carotid artery dissection. | 1 |
| 57 | 2018 | English SW | Journal of Clinical Neuroscience | Multiple cranial neuropathies as a presentation of spontaneous internal carotid artery dissection: A case report and literature review | 1 |
| 58 | 2018 | Mes M | Neurologia I Neurochirugia Polska | Hypoglossal nerve palsy as an isolated syndrome of internal carotid artery dissection: A review of the literature and a case report. | 1 |
| 59 | 2019 | Jurkiewicz MT | The Neuroradiology Journal | Hypoglossal nerve palsy due to carotid artery dissection: an uncommon presentation of a common problem. | 4 |
| 60 | 2019 | Chen Z | BMC neurology | Isolated hypoglossal nerve palsy from internal carotid artery dissection related to PKD-1 gene mutation | 1 |
| 61 | 2020 | Gordon E | Otorhinolaryngology-Head and Neck Surgery | Internal carotid artery dissection presenting as hypoglossal nerve palsy | 1 |
| 62 | 2020 | Siniscalchi A | Psychopharmacology Bulletin | Carotid Artery Dissection Induced Acute Tongue Swelling in a Cocaine User. | 1 |
| 63 | 2021 | Evan J | BMJ Case Reports CP | Dysphagia, Dysphonia and a Deviated Tongue: Diagnosing Collet-Sicard Syndrome. | 1 |
| 64 | 2022 | Sepulveda I | American Journal of Diagnostic Imaging | Isolated hypoglossal nerve palsy due to spontaneous internal carotid artery dissection: Two case reports and review of the literature | 2 |
| 65 | 2022 | Abukeshek T | Acta Raiologica Open | Hypoglossal Nerve Palsy Due to Internal Carotid Artery Dissection with Pseudoaneurysm Formation: An Unusual Presentation | 1 |
| 66 | 2022 | Kidoguchi T | Surgical Neurology International | Carotid artery stenting for spontaneous internal carotid artery dissection presenting with hypoglossal nerve palsy: A case report | 1 |
| 67 | 2023 | Kordjian HH | Ugeskrift for Laeger | Hypoglossal nerve palsy in a patient with internal carotic dissection. | 1 |
| 68 | 2023 | Shibata Y | Cureus | Extracranial Internal Carotid Arterial Dissection With Hypoglossal Nerve Palsy Caused by Cervical Self-Massage: A Case Report. | 1 |
| 69 | 2023 | Pawlukowska W | Brain Sciences | Acute Tongue Swelling as a Still Unexpected Manifestation of Internal Carotid Artery Dissection: A Case Report. | 1 |
| 70 | 2024 | Bhavsar D | Cureus | A Case of Horner's Syndrome Aiding the Diagnosis of Internal Carotid Artery Dissection (ICAD): A Life-Saving Twist of Fate. | 1 |
| 71 | 2024 | Theodorou A | European Journal of Neurology | Collet-Sicard syndrome due to cervical artery dissection disclosed by high-resolution magnetic resonance imaging | 1 |
| 72 | 2025 | Luu CP | Brain Sciences | Isolated Hypoglossal Nerve Palsy in the Setting of Concurrent Vertebral Artery Dissection and Internal Carotid Artery Dissection Plus Pseudoaneurysm: Case Report and Literature Review | 1 |
| 73 | 2025 | Bruckner JJ | Radiology Case Reports | Hypoglossal nerve palsy due to cervical ICA dissection and pseudoaneurysm: A case treated with endovascular stent placement | 1 |

Supplementary Table 3. Risk of Bias Assessment of Included Studies Using the Joanna Briggs Institute (JBI) Checklist

| Number | Study (Author, Year) | Q1 | Q2 | Q3 | Q4 | Q5 | Q6 | Q7 | Q8 |
| --- | --- | --- | --- | --- | --- | --- | --- | --- | --- |
| 1 | Bradac G,1981 | Y | Y | U | Y | U | Y | Y | U |
| 2 | Havelius U, 1982 | Y | Y | Y | Y | U | U | Y | U |
| 3 | Goodman J.M, 1983 | Y | Y | U | Y | U | U | Y | U |
| 4 | Hommel M, 1984 | Y | Y | U | Y | U | Y | Y | U |
| 5 | Goldberg H, 1986 | Y | Y | Y | Y | U | Y | Y | U |
| 6 | Mokri B, 1987 | Y | Y | U | Y | U | U | Y | Y |
| 7 | Anzola G, 1987 | Y | Y | U | Y | Y | U | Y | Y |
| 8 | Waespe W, 1988 | Y | Y | Y | Y | Y | Y | Y | Y |
| 9 | Lieschke G, 1988 | Y | Y | U | Y | Y | Y | Y | U |
| 10 | Dal Pozzo G, 1989 | Y | Y | Y | Y | U | Y | Y | Y |
| 11 | Panisset M, 1990 | Y | Y | Y | Y | U | U | Y | Y |
| 12 | Nusynowitz R, 1990 | Y | Y | U | Y | Y | Y | Y | U |
| 13 | Pumar J, 1992 | Y | Y | Y | Y | Y | Y | Y | Y |
| 14 | Sturzenegger M, 1993 | Y | Y | Y | Y | U | Y | Y | U |
| 15 | Klossek J, 1994 | Y | Y | Y | Y | Y | U | Y | Y |
| 16 | Boukobza M, 1998 | Y | Y | Y | Y | Y | Y | Y | Y |
| 17 | Lecoules S, 1999 | Y | Y | Y | Y | Y | Y | Y | Y |
| 18 | Ursekar M, 2000 | Y | Y | Y | Y | Y | Y | Y | U |
| 19 | Heckmann J, 2000 | Y | Y | Y | Y | U | Y | Y | Y |
| 20 | Guy N, 2001 | Y | Y | Y | Y | Y | Y | Y | Y |
| 21 | Pakdemirli E, 2001 | Y | Y | Y | Y | Y | Y | Y | Y |
| 22 | Shahab R, 2001 | Y | Y | Y | Y | Y | Y | Y | Y |
| 23 | Bravo-Rodríguez F, 2002 | Y | Y | Y | Y | Y | Y | Y | U |
| 24 | Bonkowsky V, 2002 | Y | Y | Y | Y | Y | Y | Y | U |
| 25 | Lindsay F, 2003 | Y | Y | Y | Y | Y | Y | Y | Y |
| 26 | Wessels T, 2003 | Y | Y | Y | Y | Y | Y | Y | Y |
| 27 | Hafkamp H, 2004 | Y | Y | Y | Y | Y | Y | Y | U |
| 28 | Dorsey S, 2004 | Y | Y | Y | Y | Y | Y | Y | Y |
| 29 | Knibb J, 2005 | Y | Y | Y | Y | Y | Y | Y | Y |
| 30 | Olzowy B, 2006 | Y | Y | Y | Y | Y | Y | Y | Y |
| 31 | Kaushik S, 2009 | Y | Y | Y | Y | Y | Y | Y | Y |
| 32 | Marin L, 2009 | Y | Y | Y | Y | Y | Y | Y | Y |
| 33 | Bezerra M, 2009 | Y | Y | Y | Y | Y | Y | Y | Y |
| 34 | Battaglia F, 2009 | Y | Y | Y | Y | Y | Y | Y | Y |
| 35 | Arnoldner C, 2010 | Y | Y | Y | Y | Y | Y | Y | Y |
| 36 | Isildak H, 2010 | Y | Y | Y | Y | Y | Y | Y | Y |
| 37 | Mathey D, 2010 | Y | Y | Y | Y | Y | Y | Y | Y |
| 38 | Freilinger T, 2010 | Y | Y | Y | Y | Y | Y | Y | Y |
| 39 | Kasravi N, 2010 | Y | Y | Y | Y | Y | Y | Y | Y |
| 40 | Stübgen J, 2011 | Y | Y | Y | Y | Y | Y | Y | Y |
| 41 | Mizutani S, 2011 | Y | Y | Y | Y | Y | Y | Y | Y |
| 42 | Peltz E, 2011 | Y | Y | Y | Y | Y | Y | Y | Y |
| 43 | Okunomiya T, 2012 | Y | Y | Y | Y | Y | Y | Y | Y |
| 44 | De Santis F, 2012 | Y | Y | Y | Y | Y | Y | Y | Y |
| 45 | Zeleňák K, 2013 | Y | Y | Y | Y | Y | Y | Y | Y |
| 46 | Riancho J, 2013 | Y | Y | Y | Y | Y | Y | Y | Y |
| 47 | Pongmoragot J, 2013 | Y | Y | Y | Y | Y | Y | Y | Y |
| 48 | Ferlazzo E, 2013 | Y | Y | Y | Y | Y | Y | Y | Y |
| 49 | Smith R, 2013 | Y | Y | Y | Y | Y | Y | Y | Y |
| 50 | Hennings J, 2014 | Y | Y | Y | Y | Y | Y | Y | Y |
| 51 | Fujii H, 2014 | Y | Y | Y | Y | Y | Y | Y | Y |
| 52 | Qi B, 2015 | Y | Y | Y | Y | Y | Y | Y | Y |
| 53 | Murakami Y, 2016 | Y | Y | Y | Y | Y | Y | Y | Y |
| 54 | Torbus-Paluszczak M,  2017 | Y | Y | Y | Y | Y | Y | Y | Y |
| 55 | Cruciata G, 2017 | Y | Y | Y | Y | Y | Y | Y | Y |
| 56 | Joshi P, 2017 | Y | Y | Y | Y | Y | Y | Y | Y |
| 57 | English SW, 2018 | Y | Y | Y | Y | Y | Y | Y | Y |
| 58 | Mes M, 2018 | Y | Y | Y | Y | Y | Y | Y | Y |
| 59 | Jurkiewicz MT, 2019 | Y | Y | Y | Y | Y | Y | Y | Y |
| 60 | Chen Z, 2019 | Y | Y | Y | Y | Y | Y | Y | Y |
| 61 | Gordon E, 2020 | Y | Y | Y | Y | Y | Y | Y | Y |
| 62 | Siniscalchi A, 2020 | Y | Y | Y | Y | Y | Y | Y | Y |
| 63 | Evan J, 2021 | Y | Y | Y | Y | Y | Y | Y | Y |
| 64 | Sepulveda I, 2022 | Y | Y | Y | Y | Y | Y | Y | Y |
| 65 | Abukeshek T, 2022 | Y | Y | Y | Y | Y | Y | Y | Y |
| 66 | Kidoguchi T, 2022 | Y | Y | Y | Y | Y | Y | Y | Y |
| 67 | Kordjian HH, 2023 | Y | Y | Y | Y | Y | Y | Y | Y |
| 68 | Shibata Y, 2023 | Y | Y | Y | Y | Y | Y | Y | Y |
| 69 | Pawlukowska W, 2023 | Y | Y | Y | Y | Y | Y | Y | Y |
| 70 | Bhavsar D, 2024 | Y | Y | Y | Y | Y | Y | Y | Y |
| 71 | Theodorou A, 2024 | Y | Y | Y | Y | Y | Y | Y | Y |
| 72 | Luu CP, 2025 | Y | Y | Y | Y | Y | Y | Y | Y |
| 73 | Bruckner JJ, 2025 | Y | Y | Y | Y | Y | Y | Y | Y |

Q1, Patient demographics clearly described;

Q2, Patient history and clinical condition clearly described;

Q3, Diagnostic methods clearly described;

Q4, Intervention or treatment clearly described;

Q5, Post-intervention clinical condition clearly described;

Q6, Adverse events or unanticipated events described;

Q7, Takeaway lessons provided;

Q8, Timeline clearly described.

Each item was rated as Yes (Y), No (N), or Unclear (U).

Supplementary table references

1. Bradac G, Kaernbach A, Bolk-Weischedel D, Finck G. Spontaneous dissecting aneurysm of cervical cerebral arteries: Report of six cases and review of the literature. Neuroradiology. 1981;21(3):149-54.

2. Havelius U, Hindfelt B, Brismar J, Cronqvist S. Carotid fibromuscular dysplasia and paresis of lower cranial nerves (Collet-Sicard syndrome): case report. Journal of neurosurgery. 1982;56(6):850-3.

3. Goodman JM, Zink WL, Cooper DF. Hemilingual paralysis caused by spontaneous carotid artery dissection. Archives of neurology. 1983;40(10):653-4.

4. Hommel M, Pollak P, Gaio J, Pellat J, Perret J, Chateau R. Paralysis of the hypoglossal nerve caused by 2 aneurysms and a dissecting aneurysm of the internal carotid artery. Revue Neurologique. 1984;140(6-7):415-21.

5. Goldberg HI, Grossman RI, Gomori JM, Asbury A, Bilaniuk L, Zimmerman R. Cervical internal carotid artery dissecting hemorrhage: diagnosis using MR. Radiology. 1986;158(1):157-61.

6. Mokri B, Piepgras DG, Wiebers DO, Houser OW. Familial occurrence of spontaneous dissection of the internal carotid artery. Stroke. 1987;18(1):246-51.

7. Anzola G, Gualandi G, Orlandini A, Scipione V. Lower cranial nerve palsy produced by internal carotid artery dilatation. Report of two cases. The Italian Journal of Neurological Sciences. 1987;8(4):375-9.

8. Waespe W, Niesper J, Imhof H-G, Valavanis A. Lower cranial nerve palsies due to internal carotid dissection. Stroke. 1988;19(12):1561-4.

9. Lieschke GJ, Davis S, Tress BM, Ebeling P. Spontaneous internal carotid artery dissection presenting as hypoglossal nerve palsy. Stroke. 1988;19(9):1151-5.

10. Dal Pozzo G, Mascalchi M, Fonda C, Cadelo M, Ronchi O, Inzitari D. Lower cranial nerve palsy due to dissection of the internal carotid artery: CT and MR imaging. Journal of computer assisted tomography. 1989;13(6):989-95.

11. Panisset M, Eidelman BH. Multiple cranial neuropathy as a feature of internal carotid artery dissection. Stroke. 1990;21(1):141-7.

12. Nusynowitz R, Stricof D. Pseudoaneurysm of the cervical internal carotid artery with associated hypoglossal nerve paralysis: demonstration by CT and angiography. Neuroradiology. 1990;32(3):229-31.

13. Pumar J, Alvarez M, Arrojo L, Otero E, Mínguez I, Vidal J. Pseudoaneurysm of the cervical carotid artery with hypoglossal and glossopharyngeal nerve paralysis. The European Journal of Medicine. 1992;1(4):246-8.

14. Sturzenegger M, Huber P. Cranial nerve palsies in spontaneous carotid artery dissection. Journal of Neurology, Neurosurgery & Psychiatry. 1993;56(11):1191-9.

15. Klossek J-M, Vandenmarq P, Neau JP, Fontanel JP. Unilateral lower cranial nerve palsies due to spontaneous internal carotid artery dissection. Annals of Otology, Rhinology & Laryngology. 1994;103(5):413-5.

16. Boukobza M, Ast G, Reizine D, Merland J. Internal carotid artery dissection causes hypoglossal nerve palsy: CT, MRI, and angiographic findings. Journal of neuroimaging. 1998;8(4):244-6.

17. Lecoules S, Coutant G, Verdalle P, Ceccaldi B, Algayres J, Daly J. Spontaneous dissecting aneurysm of the internal carotid artery. La Revue de Medecine Interne. 1999;20(5):427-30.

18. Ursekar M, Singhal B, Konin B. Hypoglossal Nerve Palsy Due to Spontaneous Dissection of the Internal Carotid Artery: Case Report. Clinical radiology. 2000;55(12):978-9.

19. Heckmann J, Tomandl B, Duhm C, Stefan H, Neundörfer B. Collet-Sicard syndrome due to coiling and dissection of the internal carotid artery. Cerebrovascular Diseases. 2000;10(6):487-8.

20. Guy N, Deffond D, Carriere N, Dordain G, Clavelou P, Gabrillargues J. Spontaneous internal carotid artery dissection with lower cranial nerve palsy. Canadian journal of neurological sciences. 2001;28(3):265-9.

21. Pakdemirli E, Usal D, Tali E. Spontaneous bilateral internal carotis artery dissection with hypoglossal nerve palsy. Computerized medical imaging and graphics. 2001;25(5):373-8.

22. Shahab R, Savy L, Croft C, Hung T. Isolated hypoglossal nerve palsy due to internal carotid artery dissection. Journal of laryngology and otology. 2001;115(7):587-9.

23. Bravo-Rodriguez F, Delgado-Acosta F, Cano-Sánchez A, Ramos-Gómez M, Ochoa-Sepúlveda J, Vida-López J. Paralysis of cranial nerves as a form of presentation of dissection of the internal carotid artery. Revista de Neurologia. 2002;34(8):754-7.

24. Bonkowsky V, Steinbach S, Arnold W. Vertigo and cranial nerve palsy caused by different forms of spontaneous dissections of internal and vertebral arteries. European archives of oto-rhino-laryngology. 2002;259(7):365-8.

25. Lindsay FW, Mullin D, Keefe MA. Subacute hypoglossal nerve paresis with internal carotid artery dissection. The Laryngoscope. 2003;113(9):1530-3.

26. Wessels T, Spitzer C, Sparing R, Klötzsch C. Isolierte Hirnnervenausfälle bei Karotisdissektionen. Der Nervenarzt. 2003;74(2):175-8.

27. Hafkamp HC, Van Der Goten A, Manni JJ. Unilateral spontaneous dissection of the internal carotid artery presenting as hypoglossal nerve palsy. European Archives of Oto-Rhino-Laryngology and Head & Neck. 2004;261(7):405-8.

28. Dorsey S, Mace S. Clinical pearls: headache and hypoglossal nerve palsy. Academic emergency medicine. 2004;11(6):668-9.

29. Knibb J, Lenthall R, Bajaj N. Internal carotid artery dissection presenting with ipsilateral tenth and twelfth nerve palsies and apparent mass lesion on MRI. The British Journal of Radiology. 2005;78(931):659-61.

30. Olzowy B, Lorenzl S, Guerkov R. Bilateral and unilateral internal carotid artery dissection causing isolated hypoglossal nerve palsy: a case report and review of the literature. European Archives of Oto-Rhino-Laryngology and Head & Neck. 2006;263(4):390-3.

31. Kaushik S, Abhishek K, Sofi U. Spontaneous dissection of internal carotid artery masquerading as angioedema. Journal of general internal medicine. 2009;24(1):126-8.

32. Marin LF, Bichuetti DB, Felício AC, Santos WACd, Oliveira FFd, Morita ME, et al. Hypoglossal nerve palsy as the sole manifestation of spontaneous internal carotid artery dissection. Arquivos de neuro-psiquiatria. 2009;67(1):107-8.

33. Bezerra MLE, Pedroso JL, Pieri A. Spontaneous carotid dissection with hypoglossal nerve palsy as residual deficit: the importance of magnetic resonance evaluation. Arquivos de neuro-psiquiatria. 2009;67(4):1109-10.

34. Battaglia F, Martini L, Tannier C. Collet-Sicard syndrome after carotid artery dissection. Revue Neurologique. 2008;165(6-7):588-90.

35. Arnoldner C, Riss D, Wagenblast J, Starlinger V, Hamzavi J-S. Tenth and twelfth nerve palsies in a patient with internal carotid artery dissection mistaken for cervical mass lesion. Skull Base. 2010;20(04):301-4.

36. Isildak H, Karaman E, Ozdogan A, Ibrahimov M, Yilmaz M. Unusual manifestations of bilateral carotid artery dissection: dysphagia and hoarseness. Dysphagia. 2010;25(4):338-40.

37. Mathey DG, Wandler A, Rosenkranz M. Hypoglossal-Nerve Palsy Caused by Carotid Dissection. Circulation. 2010;121(3):457-.

38. Freilinger T, Heuck A, Strupp M, Jund R. Hypoglossal nerve palsy due to internal carotid artery dissection. Vascular Medicine. 2010;15(5):435-6.

39. Kasravi N, Leung A, Silver I, Burneo JG. Dissection of the internal carotid artery causing Horner syndrome and palsy of cranial nerve XII. Cmaj. 2010;182(9):E373-E7.

40. Stübgen J-P. Unilateral macroglossia as sole presenting manifestation of internal carotid artery dissection. Ear, Nose & Throat Journal. 2011;90(9):434-6.

41. Mizutani S, Tsukuura R, Matsumura K, Watanabe M, Hanakawa I, Kamata T. Villaret's syndrome caused by internal carotid artery dissection. Rinsho Shinkeigaku= Clinical Neurology. 2011;51(8):608-11.

42. Peltz E, Köhrmann M. Internal-carotid-artery dissection and cranial-nerve palsies. New England Journal of Medicine. 2011;365(23):e43.

43. Okunomiya T, Kageyama T, Suenaga T. Teaching Neuro Images: Isolated hypoglossal nerve palsy due to internal carotid artery dissection. Neurology. 2012;79(4):e37-e.

44. De Santis F, Martini G, Thüringen P, Thaler M, Mani G, Steckholzer K. Internal carotid artery dissection after inferior alveolar nerve block for third molar dental care presented as hypoglossal nerve palsy. Vascular and endovascular surgery. 2012;46(7):591-5.

45. Zeleňák K, Zeleňáková J, DeRiggo J, Kurča E, Kantorová E, Poláček H. Treatment of cervical internal carotid artery spontaneous dissection with pseudoaneurysm and unilateral lower cranial nerves palsy by two silk flow diverters. Cardiovascular and interventional radiology. 2013;36(4):1147-50.

46. Riancho J, Infante J, Mateo JI, Berciano J, Agea L. Unilateral isolated hypoglossal nerve palsy associated with internal carotid artery dissection. Journal of Neurology, Neurosurgery & Psychiatry. 2013;84(6):706-.

47. Pongmoragot J, Bharatha A, Saposnik G. Pearls and Oy-sters: Carotid dissection with normal arterial lumen. Neurology. 2013;80(11):e115-e7.

48. Ferlazzo E, Gasparini S, Arcudi L, Versace P, Aguglia U. Isolated hypoglossal nerve palsy due to spontaneous carotid artery dissection: a neuroimaging study. Neurological Sciences. 2013;34(11):2043-4.

49. Smith R, Tassone P, Saada J. Collet-Sicard syndrome as a result of unilateral carotid artery dissection. Case Reports. 2013;2013:bcr2013200358.

50. Hennings JM, Höhn D, Schumann-Spaeth E, Weber F. Painless hypoglossal palsy as an isolated symptom of spontaneous carotid dissection. Journal of Stroke and Cerebrovascular Diseases. 2014;23(7):1988-90.

51. Fujii H, Ohtsuki T, Takeda I, Hosomi N, Matsumoto M. Isolated unilateral hypoglossal nerve paralysis caused by internal carotid artery dissection. Journal of Stroke and Cerebrovascular Diseases. 2014;23(8):e405-e6.

52. Qi B, Lu ZC, Wu W, Li YP. Bilateral dissecting aneurysms of the internal carotid arteries misdiagnosed as skull base tumors: A case report. Oncology Letters. 2015;10(2):931-3.

53. Murakami Y, Oda K, Konno Y, Matsumoto Y, Saito K. Successfully treated with endovascular therapy against lower cranial nerve paresis caused by spontaneous dissection of the cervical internal carotid artery: a case report. Journal of Neuroendovascular Therapy. 2016;10(1):30-5.

54. Torbus-Paluszczak M, Mucha S, Wawrzyńczyk M, Pierzchała K, Bartman W, Adamczyk-Sowa M. Hypoglossal nerve palsy in the course of dissection of the internal carotid arteries–Case reports. Neurologia i Neurochirurgia Polska. 2018;52(2):267-73.

55. Cruciata G, Parikh R, Pradhan M, Shah J, Greif E, Stein EG. Internal carotid artery dissection and pseudoaneurysm formation with resultant ipsilateral hypoglossal nerve palsy. Radiology case reports. 2017;12(2):371-5.

56. Joshi P, Bourke D. Isolated hypoglossal nerve palsy due to internal carotid artery dissection. Practical Neurology. 2017;17(3):233.

57. English SW, Passe TJ, Lindell EP, Klaas JP. Multiple cranial neuropathies as a presentation of spontaneous internal carotid artery dissection: a case report and literature review. Journal of Clinical Neuroscience. 2018;50:129-31.

58. Mes M, Palczewski P, Szczudlik P, Łusakowska A, Maj E, Gawel M. Hypoglossal nerve palsy as an isolated syndrome of internal carotid artery dissection: A review of the literature and a case report. neurologia i neurochirurgia polska. 2018;52(6):731-5.

59. Jurkiewicz MT, Stein JM, Learned KO, Nasrallah IM, Loevner LA. Hypoglossal nerve palsy due to carotid artery dissection: an uncommon presentation of a common problem. The Neuroradiology Journal. 2019;32(2):123-6.

60. Chen Z, Yuan J, Li H, Yuan C, Yin K, Liang S, et al. Isolated hypoglossal nerve palsy from internal carotid artery dissection related to PKD-1 gene mutation. BMC neurology. 2019;19(1):276.

61. Gordon E, Fanadka F, Atamna A, Gour A, Brahms-Tamir B, Kinani H, et al. Internal carotid artery dissection presenting as hypoglossal nerve palsy. Otorhinolaryngol Head Neck Surg. 2020;5:1-3.

62. Siniscalchi A, Perrotta P, Lochner P, Gallelli L. Carotid artery dissection induced acute tongue swelling in a cocaine user. Psychopharmacology Bulletin. 2020;50(1):44.

63. Evan J, Johansen M, Akst LM. Dysphagia, dysphonia and a deviated tongue: diagnosing Collet-Sicard syndrome. BMJ Case Reports CP. 2021;14(5):e243154.

64. Sepulveda I, Capizzano A, Ulloa P. Isolated hypoglossal nerve palsy due to spontaneous internal carotid artery dissection: Two case reports and review of the literature. American Journal of Diagnostic Imaging. 2022;8(3):57-.

65. Abukeshek T, Gbande P, Hamed R. Hypoglossal nerve palsy due to internal carotid artery dissection with pseudoaneurysm formation: an unusual presentation. Acta Radiologica Open. 2022;11(6):20584601221111701.

66. Kidoguchi T, Fukui I, Abe H, Mori K, Tamase A, Yamashita R, et al. Carotid artery stenting for spontaneous internal carotid artery dissection presenting with hypoglossal nerve palsy: A case report. Surgical Neurology International. 2022;13:225.

67. Kordjian HH, Petersen AG. Hypoglossal nerve palsy in a patient with internal carotic dissection. Ugeskrift for Laeger. 2023;185(15):V12220747-V.

68. Shibata Y. Extracranial internal carotid arterial dissection with hypoglossal nerve palsy caused by cervical self-massage: a case report. Cureus. 2023;15(9).

69. Pawlukowska W, Mross K, Jankowska M, Zwarzany Ł, Poncyljusz W, Masztalewicz M. Acute tongue swelling as a still unexpected manifestation of internal carotid artery dissection: a case report. Brain Sciences. 2023;13(4):603.

70. Bhavsar D, Gandhi S, Magdum R, Mushtaq I, Giri N. A Case of Horner’s Syndrome Aiding the Diagnosis of Internal Carotid Artery Dissection (ICAD): A Life-Saving Twist of Fate. Cureus. 2024;16(4).

71. Theodorou A, Lachanis S, Papagiannopoulou G, Maili M, Pachi I, Velonakis G, et al. Collet–Sicard syndrome due to cervical artery dissection disclosed by high‐resolution magnetic resonance imaging. European Journal of Neurology. 2024;31(10):e16398.

72. Luu CP, Lee B, Larson ME, Greeneway GP, Baskaya MK. Isolated Hypoglossal Nerve Palsy in the Setting of Concurrent Vertebral Artery Dissection and Internal Carotid Artery Dissection Plus Pseudoaneurysm: Case Report and Literature Review. Brain Sciences. 2025;15(3):225.

73. Bruckner JJ, Truong-Faulds T, Sharifi-Amina S, Mousa M. Hypoglossal nerve palsy due to cervical ICA dissection and pseudoaneurysm: A case treated with endovascular stent placement. Radiology Case Reports. 2025;20(11):5627-31.
